# Case of Uterine Hemorrhage, Successfully Treated by the Operation of Transfusion

**Published:** 1826-06

**Authors:** C. Waller


ART. II.—*Case of Uterine Hemorrhage, successfully treated by the Operation of Transfusion.* By C. WALLER, Esq.

TRUTH in science, like truth in religion, thrives by persecution. The proposers of the operation of transfusion have to thank its opponents for many of the converts it has already made; for the senseless hue-and-cry which has been raised against it has done more towards directing the attention of the profession to this subject, than all that we could have said or written in its favour; so true is the old adage—"Magna est veritas, et prevalebit." Such an impulse has been given to the operation, that I feel confident a fair trial will now be afforded it; and, if it be founded in *truth*, which I have good reason to suppose it is, neither the assertions, the sneers, nor the inuendoes of men who have not candour enough to recommend an improvement which they have not suggested themselves, will be able to stop its progress.

Every new remedy ought to be subjected to a full, free, and dispassionate examination, before it be admitted into general practice: but has this been done in the present instance? I answer, no: the opposition to it has not been carried on in that spirit of scientific inquiry which ought to characterise the members of a learned and a liberal profession. Most of the objectors have artfully and adroitly evaded the question altogether, contenting themselves with giving their opinion on the cases adduced, leaving the *principle* of the operation untouched. Now, is it consistent with reason and common sense to suppose that men who did not see the patients could give a more correct opinion than those who were present?—some of them, too, according to their own confession, who have *never had an opportunity of witnessing a case of a similar nature!* But granting, for the sake of argument, that it is so, still the grand question to be determined by the profession at large, is not whether these particular patients would have survived without the transfusion? but whether it *is*, or is *not*, a remedy that is calculated to be of service in cases *otherwise desperate*? That uterine hemorrhagies do every now and then prove destructive to life, none, I conceive, will have the hardihood to deny; and who, I would ask, is so likely to form a correct judgment of the probable termination of such cases as the practitioner in attendance.

Medical evidence is in its nature presumptive; it cannot be positive; and therefore, when patients recover under any particular treatment, it is quite impossible to prove to demonstration that they would have lost their lives if a different plan had been adopted. This, it is quite clear, will apply *generally*, and not to any particular cases. The language of our opponents is, in effect, this: "It is true your patients were excessively exhausted, but still it is possible they *might* have recovered if no operation had been performed. If you will only just let one of them *die*, and then recover her, we will believe you."

A great deal has been said about the *danger* of the operation, but we have no facts brought forward to prove it; whilst, on the contrary, the cases which have already occurred are sufficient to warrant an assertion that it, at any rate, is not necessarily attended with danger. But, like any other operation in surgery, it may be made a dangerous one in the hands of ignorance and inexperience; for, although it requires but a very moderate portion of skill, yet there are several *niceties* (if I may so express myself,) which are essentially necessary to be attended to, to ensure the success of the operation.

In the case which I am about to relate, the patient appeared to be as near death as it was possible for any one to be, without being actually dead.

The female was thirty-two years of age, of a very delicate and nervous habit, was exceedingly emaciated, and was so reduced by long-continued nausea and vomiting, that she had been for three weeks confined to her bed, and during the last ten days she could not even turn without assistance. I was summoned to her on the morning of the 24th April, and arrived at her house at a quarter before ten A.M., and was informed that she had experienced a few slight pains since three o'clock; that there had been a continual drain since that time, but latterly the discharge had increased: it had, in fact, run through the bed upon the floor. The patient was in a very alarming state; the pulse was only perceptible at intervals, and, when it was so, the beat was too indistinct to be accurately counted: it was, however, extremely rapid, about 140 in the minute. Both the upper and lower extremities were very cold, and she could scarcely answer any question that was put to her; her countenance exhibited that death-like appearance which it is impossible to describe, but which most of your readers must have witnessed in patients who are on the point of death. On examination, I found the hemorrhage profuse, the promontory of the sacrum rather advanced, and no part of the child below the brim of the pelvis: I therefore introduced the whole hand into the vagina, and ascertained the shoulder to be the presenting part. In such an alarming state of syncope did she appear, that I had my doubts whether the sudden emptying of the uterus would not destroy life. Finding, however, that the stimulus of the hand produced some slight uterine action, I was encouraged to proceed with the delivery, (two or three tea-spoonsful of brandy having been given her.) Under existing circumstances, there was no difficulty in turning the child, but the projecting promontory of the sacrum opposed a little resistance to the passage of the head. The placenta followed almost immediately, and the hemorrhage ceased.

The symptoms, however, of collapse were by no means relieved. The yolk of an egg was beaten up with brandy, and a small quantity given from time to time; but it failed in producing even a temporary rally. There was the high respiration in a most remarkable degree; the pulse conveyed a very feeble and fluttering sensation to the finger; the coldness of the surface was increasing, and there was a distressing state of restlessness. After waiting about three-quarters of an hour, it was very evident that we were losing ground, and I therefore determined on performing the operation of transfusion, aided by my friend Mr. Doubleday.

An obstacle, however, presented itself: the woman who had promised to supply us with blood now refused, and I had therefore to seek for some one else. After the lapse of half an hour, I returned, and found that during my absence the symptoms had been progressively increasing, and she was then in such a desperate state of exhaustion, that I must confess I scarcely conceived it possible for any thing to save her, though I determined to make the attempt.

A vein was opened in the arm of a gentleman, who kindly volunteered his blood on the occasion, and the operation was performed in the usual manner. I may mention in this place, (as we afterwards ascertained from the patient,) that at this period she could neither see, hear, nor speak; nor did she appear to feel, for, although she was naturally remarkably sensible to pain, there was no flinching when the integuments were cut through; neither had she the slightest idea of what we were about.

The first injection (thirteen drachms in quantity) produced no improvement in the pulse, beyond a temporary rally, (this, however, the brandy had failed in doing,) rendering it for a time rather more perceptible; but it is a remarkable fact, that from this time the restlessness ceased.

After waiting about five minutes, the injection was repeated (thirteen drachms), and it had the effect of rendering the pulse a little more perceptible, though not to any great extent.

In another five minutes, one ounce and a half was injected: the pulse now became tangible, beating at the rate of 124 strokes in the minute. She, however, still felt very cold; there was the high respiration, with occasional sighing; the aspect of the lips was rather improved.

In about five minutes more, fifteen drachms were injected: the pulse became a little more hurried after this injection, (140 in the minute,) but was still more perceptible; the respiration became for a few seconds a little more laborious. Her breathing was also rather stertorous, appearing as if she were in a dose; for this symptom went off if she was roused. She could at this time answer any question that was put to her.

The blood now flowed slowly from the arm of the gentleman who was supplying me: I consequently did not like to throw any more in, but had recourse to the arm of my own nephew, a healthy youth about fourteen years of age, whom I had taken with me for the purpose, and injected another fifteen drachms.* After this injection, there was a most decided improvement; the pulse became firmer, (130 in the minute,) the countenance improved in appearance, and the warmth was returning. In consequence of my washing the syringe with water that was too hot, the valve became imperfect; and I was on that account obliged to desist, which I regretted exceedingly; for, although I was quite satisfied that her improvement was sufficient to remove any apprehension of her dying from the *immediate* effects of the hemorrhage, yet, from her previously exhausted condition, and from the irritable state of the stomach, which I feared would prevent the introduction of the necessary supply of nourishment, I should have considered her ultimate recovery more certainly secured by one or two more injections, although the quantity of blood injected (eight and a half ounces) was twice as much as was employed in my first patient.† The operation finished about one o'clock, and in about an hour afterwards I left her.

In this case it is to be observed, that the introduction of the blood did not produce any temporary symptoms of distress; and this, I apprehend, was owing to the extreme slowness with which I injected it; for, from some experiments I have lately

---

* It should, perhaps, be mentioned that about half an hour elapsed between this and the preceding injection.

† Vide this Journal for October, 1825.

assisted in making, I am led to infer that the sudden and forcible injection of the blood will create some unpleasant symptoms, and may, when the animal is exceedingly exhausted, extinguish the heart's action altogether, if the quantity thus forcibly injected be large.

I have before stated, that the patient had been from time to time supplied with small quantities of yolk of egg, with brandy: a little before I left the house, the whole of this was rejected from the stomach, just in the same state that it had been swallowed; it had not been acted upon in the slightest degree. This circumstance confirms my opinion that, in these *very extreme cases*, nourishment is but of little use, as the stomach does not possess the power of assimilating it.

Eight P.M.—There is a very comfortable warmth over the whole surface of the body; pulse 140, regular, though small; her countenance is cheerful; tongue clean and moist, but her stomach so irritable that she has not kept any thing on it excepting the effervescing draught. Complains of pain in the course of the vein, which was instantly relieved by loosening the plaster, which had been bound round her arm rather tightly.

Ten P.M.—Much in the same state; arm quite easy; complains of a little uterine pain.

I shall not enter into the tedious detail of every-day symptoms. I have the satisfaction of saying that the stomach became more settled after the first twenty-four hours. The pulse, for three days, ranged from 130 to 140, but there was no other alarming symptom; and, although there were several occurrences tending to retard her recovery, such as a very uncomfortable close room, a noisy house, and a want of proper attendants; yet she was enabled to sit up for about half an hour on the seventh day, and, on my visit this morning, (twelve days after her confinement,) she was sitting up in the bed with her clothes on. Her pulse remains pretty steadily at 100, and she is enabled to take a tolerable supply of nourishment; and, under all the circumstances of the case, I consider her to be doing remarkably well. She will, I doubt not, be some considerable time before she completely regains her strength, as she was so excessively reduced by illness previous to her confinement. The wound in the arm is nearly healed.

The recovery of this patient under so many discouraging circumstances has been highly gratifying to me, and has still further strengthened my conviction that transfusion of blood in desperate cases of hemorrhage, is not that wild and visionary scheme which some men are so industriously endeavouring to make it appear.

111, *Aldersgate-street; May 5th,* 1826.

